# Using end user feedback to specify an adaptive implementation strategy

**DOI:** 10.3389/frhs.2025.1702190

**Published:** 2025-12-17

**Authors:** Taren Massey-Swindle, Julie M. Rutledge, Susan L. Johnson, Geoffrey M. Curran

**Affiliations:** 1 Department of Pediatrics, University of Arkansas for Medical Sciences, Little Rock, AR, United States; 2 Department of Family and Preventive Medicine, University of Arkansas for Medical Sciences, Little Rock, AR, United States; 3 Arkansas Children's Research Institute, Little Rock, AR, United States; 4 School of Human Ecology, Louisiana Tech University, Ruston, LA, United States; 5 Department of Pediatrics, University of Colorado Anschutz Medical Campus, Aurora, CO, United States; 6 Department of Pharmacy Practice, University of Arkansas for Medical Sciences, Little Rock, AR, United States; 7 Department of Psychiatry, University of Arkansas for Medical Sciences, Little Rock, AR, United States; 8 Central Arkansas Veterans Healthcare System, Little Rock, AR, United States

**Keywords:** adaptive implementation, end-user feedback, community-engaged dissemination and implementation, early care and education, implementation science

## Abstract

**Background:**

Adaptive implementation strategies tailor support to setting needs rather than applying a uniform approach. These strategies improve efficiency and fit, yet practical guidance on identifying decision points and tailoring variables is limited. This study collected end-user and partner input to specify decision points and tailoring variables for an adaptive implementation strategy.

**Methods:**

This study focused on the evidence-based nutrition program, Together, We Inspire Smart Eating (WISE). End users and implementation partners with prior experience in WISE were recruited in two states to participate in semi-structured interviews or focus groups designed to elicit feedback to specify an adaptive implementation strategy for WISE.

**Results:**

Qualitative input supported three crucial decisions for an adaptive implementation strategy: (1) low-intensity support, the starting point for all sites, will include leadership commitments, local champions, an implementation blueprint, classroom reminders, and task-focused facilitation at the site level; (2) assessment of response to low-intensity support will occur in October (Month 3) of the school year; and (3) sites not responding by Month 3 will receive holistic facilitation and tailored educational materials at the teacher level. Participants emphasized the universal need for facilitation at all sites, with struggling sites requiring more. They also identified tailoring variables: sites with fewer than 60% of classrooms achieving fidelity would require high-intensity support.

**Conclusions:**

This study illustrates a process for using feedback from end users and partners to define key elements of an adaptive implementation strategy. Our approach holds significant potential to specify strategies for scaling health-related evidence.

## Introduction

Implementation science aims to close the gap between evidence generation and evidence use in everyday life (1). Adaptive implementation designs help determine “who needs what” when it comes to implementation support to realize that goal (2). These designs test adaptive implementation strategies that increase or tailor implementation support in a systematic, predefined fashion and support the broader implementation science goal of optimizing strategies to fit diverse contexts and needs (3, 4). In doing so, they reflect that a uniform approach rarely fits all settings (5). Further, providing more than what is required can be costly and limit the total reach and penetration of an implementation effort through inefficient allocation of resources.

To combat the limitations of a “one-size-fits-all” approach that tends to be reflected in early implementation science studies (6), adaptive implementation strategies offer crucial decision points and tailoring variables to maximize resources. Adaptive implementation strategies allow for matching support with readiness and responsiveness. Specifically, *crucial decision points* specify when and how implementation support should be adjusted, while the *tailoring variable* is the key indicator used to guide those adjustments (2, 3). Through adaptive implementation trials, these elements are tested and refined to create reproducible guides for determining who receives what support and when in real-world practice.

To date, little guidance exists on *who* should make these crucial decisions for adaptive implementation strategies and *how* these decisions should be made. This lack of practical guidance limits researchers' and implementers' ability to design adaptive strategies that are both justified and replicable. In preparing for our enhanced non-responder trial on obesity prevention practices in early care and education settings (7), our team recognized this gap and engaged in a structured process to incorporate end-user and partner input in defining the key elements of the adaptive strategy. An *enhanced non-responder trial* is a type of adaptive implementation design that begins with an initial, standard implementation strategy for all sites and then provides additional or intensified support only to those sites that do not respond adequately to the initial strategy (3). Guided by this framework, we worked collaboratively to specify: (1) what level of initial (“low-intensity”) support all sites would receive, (2) how and when a response to those strategies would be assessed, (3) what additional strategies would be offered to non-responders, and (4) what tailoring variable would be used to guide those adaptations (4). This Brief Research Report describes this collaborative process and how end-user and partner feedback informed the crucial decision points and tailoring variables of the adaptive implementation strategy tested in the trial. This work advances implementation science by providing transparent, replicable guidance on how crucial tailoring and adaptation decisions are made, which is an essential step toward greater efficiency and contextual fit in adaptive implementation design.

## Methods

### The evidence-based innovation

Together, We Inspire Smart Eating (WISE) is an evidence-based intervention designed for early care and education (ECE) settings serving children impacted by poverty. WISE was co-designed with end users through an iterative process involving focus groups, pilot testing, and systematic refinement based on parent and teacher feedback. WISE promotes evidence-based practices (EBPs) in nutrition with the goal of improving children's diets and preventing obesity and obesity-related disease (e.g., cancer, diabetes, heart disease) (8, 9). Evidence supports benefits associated with WISE both overall and for each of its core EBPs: (1) multiple hands-on exposures to fruits and vegetables to support food acceptance (10–16), (2) role modeling by educators to allow children to observe a trusted adult interacting with novel foods (17–19), (3) positive feeding practices to support children's self-regulation and autonomy (19–21), and (4) use of a mascot to provide a familiar character linked with fruits and vegetables (22–27). Compared to usual educational practices, WISE increased fruit and vegetable intake at home for children, according to parent reports (28), as well as objective measures of carotenoid intake (29).

### Study design

To explore perceptions of WISE implementation, we drew on data from ECE educators (*N* = 82) who participated in implementations of WISE in two prior studies across two states and six sites were included in the potential participant pool. In both studies, an enhanced implementation approach (“high intensity”) was used, which is described fully in Swindle et al. (49). Survey data were used to identify ECEs with high (top quartile) and low (bottom quartile) ratings of WISE feasibility, which informed purposive sampling for semi-structured interviews. We selected feasibility as the target outcome for sampling to capture perceptions of the innovation, recognizing that fidelity can remain high even when attitudes toward implementation are neutral or negative, which may undermine long-term sustainment (30). To broaden perspective, we also interviewed center directors (*N* = 4, 80% of those eligible) and food service staff (*N* = 8, 75% of those eligible) across the six sites, creating a multi-stakeholder view of implementation. The study was reviewed and approved by the Institutional Review Board (IRB) at the University of Arkansas for Medical Sciences. Consent for interviews was provided verbally in accordance with IRB-approved procedures.

### Measures

The integrated-Promoting Action on Research Implementation in Health Services (i-PARiHS) framework guided the development of the interview guide. The i-PARiHS framework posits that core components of successful implementation include *innovation* (the EBPs), *recipients* (individual and collective), *context* (inner and outer), and *facilitation* (31). In line with these domains, questions about context explored how staff recognized peers who were excelling or struggling with WISE and how the implementation team identified centers that needed additional support. Questions about innovation focused on perceptions of the value and cost-effectiveness of possible implementation strategies. Questions about the recipients examined which WISE resources were used most and why by each end-user group. Finally, questions about facilitation asked participants to prioritize the most crucial implementation supports if only a few could be provided. According to i-PARiHS, successful implementation takes place when facilitation promotes the acceptance and use of an innovation based on the recipients' needs and the nature of the context. Interviews explored how these elements influenced WISE delivery. See example questions in Table 1. After the interview, the interviewer provided a summary of the interview to the participant with the invitation to correct any inaccurate conclusions, provide additional detail, and clarify any comments, i.e., participant validation (32) and member checking (33). All the participants were paid a $50 check in accordance with IRB-approved procedures. Data collection continued until saturation was achieved, defined by consensus among the coders as the point when no new ideas emerged from additional interviews (34).

**Table 1:** Sample interview questions by i-PARiHS construct.

i-PARiHS constrct	Sample interview questions
Context	How long was it after WISE training before you knew which of your peers liked WISE? And which were struggling? How would the WISE team know who at your center needed our help?
Innovation	I would like to share the cost per classroom of each of the various implementation strategies we used to support you and your centers in WISE. Which seems like a good value? Which are too costly for the value you received?
Recipients	Which of the resources provided to you to support WISE implementation did you use most and why?
Facilitation	If we could have only given you three implementation supports, what would have been most crucial and why?

### Participants

In total, we interviewed 11 teachers and 4 administrators and conducted two focus groups with 8 food service staff. All the participants were female, and 83% were Black. Of the six sites, two were located in a town with a population of 22,330; the remainder were in an urban city with a population of 204,774. Further, five of the six sites were Head Start locations serving children impacted by poverty; one site was a public Pre-K serving children across a variety of income levels. Participants were evenly split across the two studies and the states from which they were drawn. This range of participants allowed us to examine perspectives from multiple roles and organizational levels involved in WISE implementation.

### Analyses

Interviews were transcribed verbatim using a third-party, Health Insurance Portability and Accountability Act (HIPPA)-compliant transcript service. We used a rapid, directed content analysis with a deductive lens (50), an approach well-suited for the focused objective of our interviews to identify the most useful aspects of WISE implementation support for specifying the adaptive implementation strategy. Excel-based matrices were used to organize the data, as is common in rapid analytic approaches (35, 36). Coders (*N* = 3) met to establish a codebook with shared meaning and consensus of code application using four interviews. Afterward, coding was independent with regular consensus meetings to reduce bias, ensure reliability and validity, and expand the codebook as needed. The team then met to review the coded data, identify recurring patterns, and develop consensus on the alignment of codes with the deductive purpose (i.e., define critical decisions and tailoring variables). Through iterative discussion and data comparisons, preliminary decisions were proposed, which were confirmed against the full dataset with consideration of supporting and countering perspectives.

## Results

### Low-intensity support

Consistent with the conceptualization of an adaptive implementation strategy, input from participants suggested that a “light touch” of support would be sufficient to support success for many classrooms and sites. These low-intensity strategies addressed the i-PARiHS constructs of *context* (i.e., a formal commitment from leadership, a local champion) and *facilitation* (task-focused monthly technical support) to provide practical problem-solving. The commitment from leadership was consistently nominated among the important strategies to keep by participants, while the local champion was perceived to be a high-value strategy relative to the cost (See Table 1, Facilitation sample question). Incentives were infrequently nominated as a top strategy and eliminated because participants perceived the incentives as: (1) potentially undermining the relationship between the team and the end users and (2) encouraging extrinsic motivation instead of intrinsic motivation.

Table 2 provides illustrative quotes that support the inclusion of strategies in the low-intensity, foundational package. The quotes show that leadership commitment inspired enthusiasm and collective purpose among teachers; the implementation blueprint offered clarity about expectations; and the local champion served as a trusted liaison who kept communication flowing between the WISE team and centers. Participants also described how visual cues, such as the cutting board, helped keep lessons salient in daily routines and how task-focused facilitation provided timely, hands-on problem-solving when barriers arose. Together, these examples demonstrate that partners valued strategies that were practical, visible, and immediately useful. These features were suggested to support the *recipients’* engagement with the *innovation*.

**Table 2:** Salient quotes for partner input on low-intensity strategy.

Strategy	Partner input
Formal commitment from leadership	“And they (leadership) believed that it was making a difference in the lives of the children. So, if you see somebody that's really excited about what they're doing. The, you know, they're energized there. You know, just, ‘Hey, guys. We're gonna do this. We can do this to make the children better. We're going to make them happier. We're gonna make them healthier.’ That's all I needed.”—Teacher
Implementation blueprint	“Maybe the blueprint … them telling us exactly what they kind of expected of us and what they needed done …. helping them on the same page. That was where they told us, you know. They gave us the supplies and told us you know what they expect.”—Director
Local champion	“For the sites, I would say the number one probably helpful would be the champion because that was the person, the one what would take the information back and forth from the meetings to the sites. So, for the sites, I would say that probably having that champion be the liaison between the two was probably the most helpful.”—Administrator
	“The site champions … was most helpful. I'm very comfortable with my champion, and, I mean, it's really easy to get to her because she was just like, right there. So I'm like, rather than like emailing and not being able to have like an actual like face-to-face conversation. It's nice to have a conversation with the site champion and be like, ‘Hey, I don't know how to do this and this.’ … Or like getting ideas from her. You know her telling me what she's doing in the classroom, what's working for her. It was it's nice to have that like actually at the site.”—Teacher
Cutting board reminder	“The staff, our staff like to have visuals, and providing visuals works well with them. And to be honest, I have to have a visual, you know, to keep me on-on task.”—Administrator
Task-focused facilitation	“I think the thing that was the most helpful was the coaching along the way. Because I think it was real important that when we ran into obstacles, that, you know, we could come into you and say, ‘Hey, this isn't working. You know, we need some-some different ideas.’ I remember, we'd had an issue with some of our teachers wanting to go, like, directly by the book. And they got really upset when we were limited as to what we could order, and so they would get very upset if we couldn't get the exact things that they needed. And they didn't wanna, you know, kinda change things up. I remember we came to you and said, ‘Can you help us?’ And so you guys were always there as a resource, you know.”—Director
	“And then, like I said, anytime we had an issue, I mean, it was—didn't take—it was the same day, we would try to have it resolved. You know, when we'd call and say, ‘Help,’ you know, you guys were right there to help us.”—Food Service Staff

### Timing of response assessment

In addition to establishing the foundational package, input from the participants was used to inform the timing of the assessment of response to the low-intensity support, as well as the tailoring variable to determine response. For the timing of the assessment, suggestions ranged from 0.5 to 4 months into the school year, with the majority stating that October (approximately 2.5 months into the school year) would allow a good sense of which classrooms needed additional support. A center director stated:

“At first it was bumpy because they weren’t getting the materials that they needed…so there was a lot of adjusting that we had to do. But then once you would see the projects happening, the-teachers facilitating it. So I’d say maybe about three months after we kinda got a hang of things.”

A teacher also reflected:

“But after a while, of getting used to it, then I heard some other teachers being like, ‘Okay, it’s actually not so bad. It just takes some time to ease into’. And I say a few months …. maybe, October, November. Because that way, that gives the teacher some time at the beginning of the year to get settled into a routine. But it’s not too late to where you've kind of ruined the whole concept of building up WISE in the machine. So I would think, probably, October, November.”

An administrator supported these perspectives, stating,

“You know, right off the bat, you know, I knew we were struggling a little bit. But, you know, I—that’s why I said I think probably by—within October or November, everybody had a good feel for it.”

Taken together, participants emphasized that assessment of response to the low-intensity support should occur after an initial adjustment period but early enough to identify classrooms that may benefit from additional assistance. Across roles, there was consistent agreement that October or November, approximately 2–3 months into the school year, represents an optimal window for determining whether classrooms are responding adequately or may need to transition to higher-intensity support. This timing reflects a sensitivity to *context*, recognizing that educators need an initial stabilization period within their organizational and classroom routines before meaningful assessment of implementation can occur.

### Tailoring variable to assess response

Input on the tailoring variable emphasized the importance of seeing implementation firsthand. Specifically, participants suggested observing teachers’ behaviors during the lesson (use of inappropriate practice, use of lesson plan, willingness to welcome WISE staff and talk with them), teachers' attitudes during the lesson (e.g., excitement, enthusiasm, comfort), and children's behaviors (e.g., awareness of Windy, excitement based on lesson cues). A center director recommended:

“I think just from observing them actually do one of the lessons, you know, you can typically identify, you know, who’s comfortable, and who’s not, um, with certain topics. And so if someone’s not real comfortable … I would identify them maybe as needing some additional support, or if you had some that just kinda didn’t seem interested. And-and like I said, I think most of it, uh, would be from observation.”

Teachers provided a similar perspective on the approach to identifying who may need additional help:

“See if the teacher was like willing to talk about WISE. … if they were just like, not welcoming. I think that that teacher would need additional support. I'm looking around the room to see if anything WISE related was on the walls or just presented anywhere in the room. If you had conversations with kids and like, ‘What has Windy the the owl brought to you?’ The kids can talk about it.”

An agency administrator also described how witnessing activities firsthand assured her that the implementation of WISE was going well or helped to identify someone who may need extra support:

“Whatever the activity …. That you-you could just see it happen. Even with the WISE puppet, you would see it in there at circle time or group time being pulled out as well as during the activities. The children would be able to speak about it …. you know, whenever I'm out in the sites, I get to be able to see it … and if the teacher, you know, wasn't excited at least while they were introducing the-the activity to the-the children then that would bring some concern to me as well.”

Across roles, participants consistently emphasized that identifying which classrooms may need additional support (i.e., assessing response via a tailoring variable) required seeing implementation firsthand, rather than relying on self-report or documentation. Their feedback directly informed the decision to operationalize the tailoring variable through onsite fidelity observations (51) of the four WISE EBPs and to set a threshold under which a teacher would be deemed to require a shift from low-intensity to high-intensity strategies (i.e., scoring below fidelity for two or more practices). Observing lessons in person was viewed as the most valid and practical way to assess teacher engagement, comfort, and use of the WISE materials and EBPs.

### High intensity

The strategies in the high-intensity implementation package reflect input from participants on the best ways to support teachers who do not respond to the low-intensity support. That is, if the direct fidelity assessment suggested inadequate use of evidence-based practices, the addition of high-intensity support strategies would be warranted. These strategies include tailored educational materials and holistic, individualized facilitation. Tailored educational materials provided rationale and examples of how to enact WISE EBPs in the ECE classroom; educators received educational materials on the practices in which they struggled the most. Holistic facilitation differed from the task-focused facilitation of the low-intensity strategy by expanding to build rapport with the teachers and to work directly with them on their practices in the classroom (vs. helping to troubleshoot general barriers directly). This approach reflects the *facilitation* domain of i-PARiHS, emphasizing individualized coaching to enhance adoption of the *innovation* within the classroom context when *recipient*-level barriers are more complex.

Table 3 provides supportive quotes for the inclusion and specification of the high-intensity strategies. Teachers described the tailored materials as concrete reminders that helped them internalize WISE concepts and adapt the language for their classrooms, while administrators emphasized their usefulness in reinforcing priorities within centers. Across roles, participants noted that individualized facilitation became increasingly effective as trust developed; the coaching relationship evolved from initial hesitation to genuine collaboration. Teachers reported feeling encouraged and supported rather than evaluated, and administrators observed that this ongoing, responsive coaching helped maintain motivation and consistency in implementing WISE.

**Table 3:** Salient quotes for partner input on high-intensity strategy.

Strategy	Partner Input
Tailored educational materials	“Well, one thing that I can't say was challenging for me was making sure I didn't use the terminology and sentences and phrases that I was used to using that I grew up with. You know, I had, I had gotten a little better with it. But things will still come out, you know, like, ‘You need to you need to try this before you get this.’ …. We only have so much time to eat. So if you're going to eat this. I need you to eat it, you know. But then I had to just let them eat whatever they want to eat, whenever they want to eat it, however they want to eat it.”—Teacher
	“Visuals are always … I mean, we know that when there's something important that we want teachers to remember, we always have ‘em post those types of things on their, um, bulletin board.”—Director
Holistic, individualized facilitation	“I really think that once they got used to the idea of the coaching, it really helped them. But again, like I said, they had to kinda ease into that and understand that you weren't there to get ‘em in trouble or to say, ‘You're not doing this right,’ or anything like that. That you were really there for them, and to help them, you know, get this pulled together. So I think that was probably the most beneficial. At first, I don't know how receptive they were. And then I think as time went on, you know, they saw that this was fun. And the kids really enjoy it. And they kinda bought into it, I guess, as more—I'm sayin' as the year went on. So I think that probably, um, increased with all the coaching that you guys gave them.”—Administrator
	“It was still good to know that somebody appreciated what you were doing and that you know they encourage you, ‘Hey, that's a good thing you're doing.’ Sometimes as teachers we don't get that. And that really inspired me to do more, not only doing the WISE sessions, but in in the other areas in the classroom. It helps, you know … Even though it got easier for us to do it, they still never, you know, they never backed up. They were always still there. ‘Can I help you with this; can I help you with that? Do you have questions?’ It was always, ‘Do you have a concern? How do you feel about what you're doing? Do we need to talk about this? Do you need any more materials?’ I mean, it never stopped. It was like from the beginning when we were so nervous and anxious about it until you know right on up until we didn't go back to school anymore.”—Teacher

All decisions about crucial decision points and tailoring variables are summarized in Table 4. This table consolidates how partner input was translated into the final adaptive design, showing the logic that guided each element of the implementation strategy. It highlights when responses should be assessed, how fidelity observations function as the tailoring variable, and which supports are triggered for classrooms needing additional assistance. Together, these decisions reflect a systematic approach that balances feasibility with responsiveness, ensuring that implementation support is both efficient for most sites and sufficiently intensive for those requiring more hands-on guidance. Table 4, therefore, provides a concise overview of the full adaptive strategy that emerged from end-user and partner input.

**Table 4:** Adaptive implementation strategies: decisions on design features.

Design feature	Definition	Decision
Crucial decision points	Which strategies to begin the study with (i.e., low-intensity)	Formal commitment with leadership; implementation blueprint; local champion; cutting board with reminders of WISE EBPs; task-focused monthly facilitation
	How and when response is measured	October
	What strategies are given to non-responders (i.e., high-intensity)	Tailored educational materials; holistic, individualized facilitation
Tailoring variables	Measurement to identify non-responders and inform strategy intensity	Observed fidelity to WISE EBPs

EBPs, evidence-based practices.

## Discussion

The use of adaptive implementation designs is increasing to evaluate implementation strategies that provide more resource-intensive implementation support to systems and implementers that do not respond to a basic level of support (3). This trend reflects a broader movement in implementation science toward tailoring and resource optimization, matching strategy intensity to site needs rather than applying uniform support (37). Several decisions are key in designing adaptive trials that will have important implications for the use of the adaptive implementation strategy in the real world (2). Engaging implementation partners in defining the decision points of adaptive trials remains uncommon, particularly in early childhood contexts where implementation infrastructure is often limited (38, 39). Within implementation science, participatory and co-design approaches are increasingly recognized as key to improving the relevance, feasibility, and sustainability of strategies (40), yet their integration into adaptive designs remains limited. Prior studies in health and education settings have demonstrated that partner-driven adaptation can improve the feasibility and acceptability of implementation strategies (41, 42). Our findings extend this work by showing how partner input can be systematically embedded at multiple decision points to ensure adaptive implementation designs are contextually grounded and operationally feasible.

Specifically, our team used input from ECE partners with prior experience with our innovation and implementation strategies to define a tailoring variable, determine the timing of the measurement of response, and generate low- and high-intensity strategies. Low-intensity support was specified to support contextual-level logistics and leadership support for implementation, focusing on creating a supportive *setting* for implementation. High-intensity support added tailored, holistic implementation facilitation and education at the implementer level, adding an explicit focus on directly supporting the *people* doing the implementation. Input from ECE partners informed the decision to base the tailoring variable on classroom observations and to measure response 3 months into the school year. Our work illustrates that input from relevant implementation partners is a promising approach to the design of adaptive implementation strategies.

Both the low-intensity and high-intensity strategies in our adaptive implementation package have facilitation at the foundation. This reflects that the focus and targets of facilitation can vary (43). Task-oriented facilitation delivers technical and practical support (43). Reflective of this focus, task-focused facilitation was designed to focus on context-level barriers and monthly contact with leadership and champions in our low-intensity strategy. However, the holistic facilitation in the high-intensity strategy was designed to encourage the development of shared understanding, interconnected networks, and personal growth (43). According to the i-PARiHS framework (44), which guided our study, effective implementation requires diverse facilitation strategies based on the nature of the innovation, the context, and the needs of the recipients. This aligns with a central tenet in implementation science that facilitation is a dynamic, context-dependent process, not a static intervention. Thus, although it is not yet common in implementation practice, facilitation as a strategy may need to differ in systematic and replicable ways to support implementation while optimizing resources. Our adaptive implementation strategy illustrates the clear specification of the targets of each type of facilitation. This distinction has important implications for scaling and sustainability. Task-focused facilitation may be more readily scaled, whereas holistic facilitation requires personnel with advanced relational and reflective skills, suggesting the need for differentiated training and resource allocation across contexts. Future efforts could test tiered models of facilitation to determine which elements can be delivered by internal staff vs. those that require external expertise and which are most predictive of successful scale and sustainment (43, 45).

Building on these insights, we also recognize several limitations that should inform the next phase of research. One limitation is that ECE staff reported they did not feel comfortable or informed to weigh in on cost-related information, frequently citing that it was beyond their role (even center directors). Thus, input on the design of our adaptive strategy was not closely connected to a payer perspective. Future studies that engage end users in the design of adaptive implementation strategies may benefit from the inclusion of potential payers' input. In addition, the qualitative sample was small and drawn from individuals with prior experience implementing WISE and with the research team, which may have contributed to favorable perceptions. Despite these limitations, the inclusion of multiple perspectives (i.e., teachers, kitchen staff, center directors), each with a unique role in the implementation process, strengthened the validity of our findings.

## Conclusions

This study advances adaptive implementation research by providing a systematic process for integrating end-user and organizational partner input at multiple decision points in the design of an adaptive strategy, which reflects the increasing recognition that one size does not fit all when it comes to implementation strategies (4, 46). By demonstrating how partner insights can inform the specification of timing, tailoring variables, and intensity of facilitation, this work offers a replicable model for developing adaptive implementation approaches in other community and educational settings. Community contexts broadly, and the ECE setting specifically, often operate under resource constraints where optimizing personnel and financial resources is a crucial requirement (46–48). Our findings illustrate *how* context-sensitive, partnership-driven adaptation can make implementation supports both resource-efficient and responsive. Our ongoing enhanced non-responder trial will test whether this co-designed adaptive strategy improves implementation outcomes and cost-effectiveness relative to static approaches (7). In so doing, this work contributes to implementation science's broader aim of developing and testing reproducible, contextually grounded methods for improving implementation support.

## Data Availability

The datasets presented in this article are not readily available because the data cannot be fully anonymized. Requests to access the datasets should be directed to Taren Massey-Swindle at tswindle@uams.edu.
